# *Candida* spondylodiscitis: a systematic review and meta-analysis of seventy two studies

**DOI:** 10.1007/s00264-023-05989-2

**Published:** 2023-10-04

**Authors:** Siegfried J. Adelhoefer, Marcos R. Gonzalez, Angad Bedi, Arne Kienzle, Henrik C. Bäcker, Octavian Andronic, Daniel Karczewski

**Affiliations:** 1 Center for Musculoskeletal Surgery, Department of Orthopaedic Surgery, Charité - Universitätsmedizin Berlin, Charitéplatz 1, 10117 Berlin, Germany; 2 Department of Orthopaedic Surgery, Musculoskeletal Oncology Service, Massachusetts General Hospital - Harvard Medical School, 55 Fruit Street, Boston, MA 02114 USA; 3 Department of Orthopaedic Surgery, University Medical Center Groningen, Hanzeplein 1, 9713 Groningen, Netherlands; 4 Department of Orthopaedic Surgery, Auckland City Hospital, 2 Park Road, Auckland, 1023 New Zealand; 5 Department of Orthopedic Surgery, Balgrist University Hospital, Forchstrasse 340, 8008 Zurich, Switzerland

**Keywords:** *Candida albicans*, *Candida tropicalis*, Immunosuppression, Spine infection, Drug use

## Abstract

**Objectives:**

Knowledge of *Candida* spondylodiscitis is limited to case reports and smaller case series. Controversy remains on the most effective diagnostical and therapeutical steps once *Candida* is suspected. This systematic review summarized all cases of *Candida* spondylodiscitis reported to date concerning baseline demographics, symptoms, treatment, and prognostic factors.

**Methods:**

A PRISMA-based search of PubMed, Web of Science, Embase, Scopus, and OVID Medline was performed from database inception to November 30, 2022. Reported cases of *Candida* spondylodiscitis were included regardless of *Candida* strain or spinal levels involved. Based on these criteria, 656 studies were analyzed and 72 included for analysis. Kaplan-Meier curves, Fisher’s exact, and Wilcoxon’s rank sum tests were performed.

**Results:**

In total, 89 patients (67% males) treated for *Candida* spondylodiscitis were included. Median age was 61 years, 23% were immunocompromised, and 15% IV drug users. Median length of antifungal treatment was six months, and fluconazole (68%) most commonly used. Thirteen percent underwent debridement, 34% discectomy with and 21% without additional instrumentation. Median follow-up was 12 months. The two year survivorship free of death was 80%. The two year survivorship free of revision was 94%. Younger age (*p* = 0.042) and longer length of antifungal treatment (*p* = 0.061) were predictive of survival.

**Conclusion:**

Most patients affected by *Candida* spondylodiscitis were males in their sixties, with one in four being immunocompromised. While one in five patients died within two years of diagnosis, younger age and prolonged antifungal treatment might play a protective role.

**Supplementary Information:**

The online version contains supplementary material available at 10.1007/s00264-023-05989-2.

## Introduction

*Candida albicans* is the most common pathogen involved in bloodstream infections [1], with current investigations raising concerns on increasing numbers of multidrug-resistant strains as a potentially new global health threat [2–4]. Despite its importance, limited remains known on spondylodiscitis caused by *Candida* spp. [5]. Preliminary findings indicate that *Candida* spondylodiscitis affects high-risk cohorts, including those with prior IV drug abuse, obesity, and diabetes, oftentimes resulting in fatal outcomes [6, 7].

Importantly, diagnostical and therapeutical approaches among this uniquely challenging cohort remain controversial, given limited patient numbers in existing investigations [5]. In fact, to the best of our knowledge, all present investigations are case reports or smaller case series. Moreover, these limited reports on *Candida* spondylodiscitis have short-term follow-up only [8, 9], while limiting their analysis to certain spinal levels [10], the presence of instrumentation [11, 12], immunosuppressive patients [13], or certain *Candida* strains [14–16]. This is critical, as it reduces the number of cases in an already limited cohort of patients.

Given its increasing importance, controversial approaches to diagnosis and treatment, and limited patient numbers in existing reports, this review is the first to systematically summarize all cases of *Candida* spondylodiscitis to date. In addition to analyzing baseline demographics, and identifying potential patients at risk, we aimed to summarize current diagnostical and therapeutic management, together with the outcome and prognostic factors.

## Methods

This systematic review was based on PRISMA guidelines [17, 18] and registered in the PROSPERO [19] International prospective register of systematic reviews (CRD42022365441). Data collection was performed from October 17, 2022, through November 15, 2022. A re-run prior to the final analysis was performed to include any studies that may have been published during the data collection phase.

We analyzed a total of five databases (PubMed, Web of Science, Embase, Scopus, and OVID Medline) using the search syntax “albicans” [Title/Abstract] AND (“spondylodiscitis” [Title/Abstract]) with variations depending on the unique syntax of each database (*Supplementary Table* *1*). If available, we also included articles from the “Similar articles”-tools from PubMed to increase the scope of articles screened [20]. Articles that were not available as a full-text English version were excluded unless an English abstract was available yielding sufficient data for analysis.

We included articles reporting microbiology-confirmed cases of *Candida* spondylodiscitis of any segment of the spine. No restrictions were made based on the year of publication, status of immunocompetency, or length of follow-up. PICO criteria [21] as a mean of evidence-based analysis were followed throughout: we included articles reporting patients with confirmed *Candida* spondylodiscitis who underwent diagnostic and therapeutic management. No comparator or control group was given and the primary outcome of interest was overall survival (*Supplementary Table* *2*). Articles were excluded if they (1) were non-original articles, (2) were solely reporting osteomyelitis without involvement of the disc, (3) were not involved human subjects, and (4) reported spondylodiscitis caused by other infections agent(s). After removal of duplicates, titles and abstracts were screened for inclusion eligibility, and if deemed suitable analyzed as a full text.

We collected baseline demographics and information on past history, including age, sex, immunocompetency, presence of IV drug abuse, duration and types of clinical symptoms, previous spinal surgeries, instrumentation status, and affected spinal segment. Comorbidities were analyzed based on the age-adjusted Charlson Comorbidity Index (CCI) [22, 23]. In terms of diagnostical workup, we collected data items on CT-guided biopsy prior to initiating antifungal treatment, leukocyte count, erythrocyte sedimentation rate (ESR), and C-reactive protein (CRP), as well as microbiology results. Lastly, we gained data on the treatment regimen including antifungal agents and operative procedures.

Outcome parameters included length of hospital stay (LOS), perioperative complications, number of revisions, final recovery status, and mortality. Partial recovery was defined as continued clinical symptoms or permanent neurologic damage, whereas full recovery was defined as resolution of all initial symptoms at the most recent follow-up.

All results were presented as (1) overall cohort, (2) separated by *Candida albicans* versus *non-albicans*, and (3) based on survival at the last follow-up. Categorical variables were presented as absolute and relative values, and continuous variables as median and interquartile ranges. For comparison between groups, we used Fisher’s exact test for binary data to compare frequencies of side effects in two groups and Wilcoxon’s rank sum test to compare continuous data. A *p*-value of < 0.05 was considered significant. Kaplan-Meier survivorship analyses were performed, and differences in survival calculated based on log-rank tests.

## Results

**Fig. 1:**
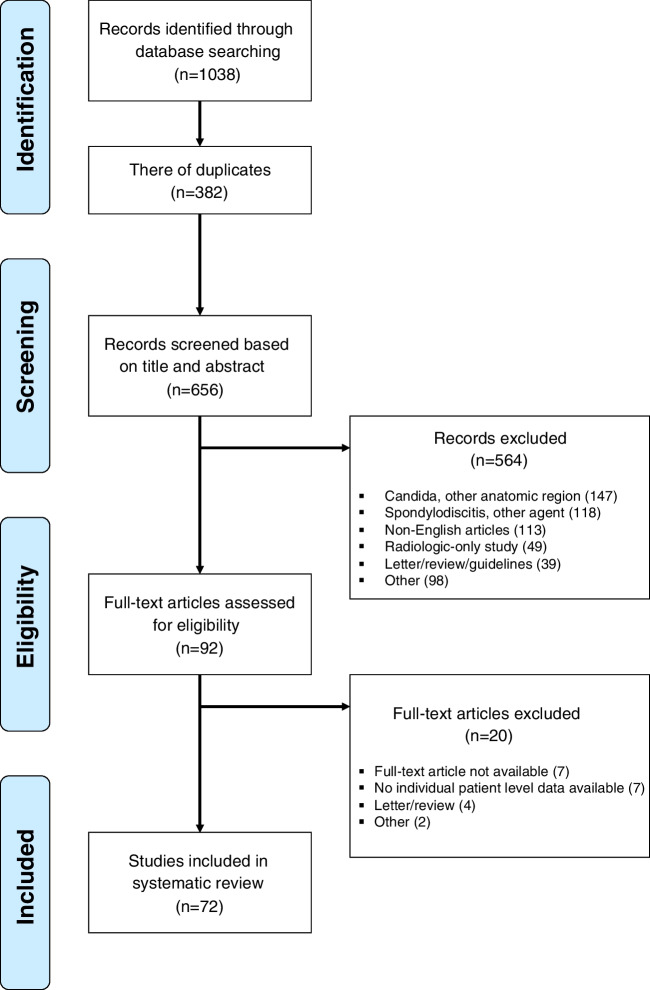
PRISMA flow chart

After removing 382 duplicates from the initial 1038 studies identified in the search process, 656 articles were analyzed for title and abstract. Following further detailed exclusion criteria, we analyzed 92 articles as full texts, of which 72 were finally included (Fig. 1).

The final cohort consisted of 89 patients with a median age of 61 (Table 1, Table 2). Median age-adjusted CCI was 3, 15% had a history of IV drug use, and 23% of patients were immunocompromised. Nearly all patients presented with back or neck pain (99%), whereas fever and weakness were present in 19% only. Symptoms lasted for an average of nine weeks until the final diagnosis was made. Median leukocyte count was 8×10^9^/mL, CRP was 3 mg/dL, and ESR was 65 mm/h. CT-guided biopsy was performed in 53%, with *Candida albicans* (60%) identified as the most common pathogen. Empiric antibiotic treatment prior to definite diagnosis was administered in 41% of cases. Antifungal monotherapy was given in 58%. The most commonly used antifungal agents included fluconazole (68%), amphotericin B (38%), and echinocandins (26%). The median length of antifungal treatment was six months. Surgical intervention was performed in 68%, including 34% undergoing instrumented discectomy. At a median follow-up of 12 months, 3% developed sepsis, 6% underwent revision, and 12% died of disease. The two year survivorship free of death was 80% (95% CI, 62 to 98%), and the two year survivorship free of revision was 94% (95% CI, 84 to 100%).

**Table 1:** Overview of 89 patients with *Candida* spondylodiscitis identified in 72 studies

Study	No. of patients	Age (range)	Sex	Spinal level	*Candida* spp.	Medical therapy	Surgical therapy	Length of treatment, months (range)	Follow-up, months (range)
Shaikh et al., 1980 [24]	1	67	m	L1-L2	*C. albicans*	Amphotericin B	None	1.38	4
Hayes et al., 1984 [25]	1	67	m	L1-L2	*C. tropicalis*	None	None	NA	NA
Kashimoto et al., 1986 [26]	1	50	m	T7-T8	*C. tropicalis*	None	Debridement	NA	21
Herzog et al., 1989 [27]	1	88	m	L4-L5	*C. tropicalis*	Ketoconazole, amphotericin B	None	2.3	6
Hennequin et al., 1996 [28]	2	52–61	1 m 1 f	1 T10-T11 1 L3-L4	2 *C. albicans*	Fluconazole	Debridement	6	17–47
Rieneck et al., 1996 [29]	1	56	m	L3-L4	*C. albicans*	None	None	6	NA
Munk et al., 1997 [30]	1	67	m	L2-L3	*C. albicans*	Amphotericin B	None	NA	NA
Godinho de Matos et al., 1998 [31]	1	45	m	L5-S1	*C. albicans*	Fluconazole, amphotericin B	None	NA	NA
Rössel et al., 1998 [32]	1	20	f	T11-T12	*C. albicans*	Fluconazole, amphotericin B	None	8.5	12
Derkinderen et al., 2000 [33]	1	32	f	T7-T8	*C. albicans*	Fluconazole, amphotericin B, flucytosine	None	3	11
Parry et al., 2001 [34]	3	19–64	2 m 1 f	3 L4-L5	*C. albicans*	Fluconazole, amphotericin B, flucytosine	Debridement	11–13	3–8
Sebastiani et al., 2001 [35]	1	67	m	T8-T9	*C. tropicalis*	Fluconazole	None	3.33	NA
Karlsson et al., 2002 [36]	1	76	f	L2-L3	*Candida* spp. (not specified)	None	Debridement, discectomy with instrumentation	NA	36
Tokuyama et al., 2002 [13]	1	44	f	T12-L1	*C. albicans*	Fluconazole, itraconazole	Debridement, discectomy without instrumentation	NA	NA
Torres-Ramos et al., 2004 [37]	1	69	f	T8-T9	*C. tropicalis*	Amphotericin B	Debridement, discectomy with instrumentation	2.76	12
Ugarriza et al., 2004 [38]	1	70	m	T8-T9	*C. albicans*	Fluconazole	Debridement, discectomy without instrumentation	4.75	36
Chia et al., 2005 [39]	2	50–63	2 m	1 C5-C6 1 T7-T8	1 *C. albicans* 1 *C. tropicalis*	Fluconazole, amphotericin B	Debridement, discectomy without instrumentation	3.75–4.25	12
Pemán et al., 2006 [40]	1	62	m	T5-T6	*C. krusei*	Voriconazole, caspofungin	None	6	8
Kroot et al., 2007 [41]	1	28	m	L5-S1	*C. albicans*	Fluconazole	None	NA	NA
Yang et al., 2007 [42]	1	79	m	L2-L3	*Candida* spp. (not specified)	None	Debridement, discectomy without instrumentation	NA	NA
Moon et al., 2008 [43]	1	64	f	C5-C6	*C. albicans*	Fluconazole, amphotericin B	Debridement, discectomy with instrumentation	6.5	6
Schilling et al., 2008 [14]	1	58	m	NA	*C. krusei*	Fluconazole, voriconazole, posaconazole, amphotericin B, caspofungin	Debridement, discectomy with instrumentation	13	19
Cho et al., 2010 [11]	1	70	f	L5-S1	*C. parapsilosis*	Fluconazole	Debridement, discectomy without instrumentation	4	4
D’Agostino et al., 2010 [44]	4	53–74	2 m 2 f	1 C6-C7 1 T12-L4 1 L1-L2 1 L2-L3	3 *C. albicans* 1 *C. glabrata*	Fluconazole, amphotericin B	Debridement	2.07–8.28	NA
Rachapalli et al., 2010 [45]	1	48	f	T6-T7	*Candida* spp. (not specified)	Fluconazole, flucytosine	None	1.84	NA
Erné et al., 2011 [46]	1	72	NA	L1-L3	*C. albicans*	Fluconazole	Debridement, discectomy with instrumentation	NA	12
Palmisano et al., 2011 [47]	1	48	f	L3-L4	*C. sake*	Fluconazole	None	Ongoing	25
Werner et al., 2011 [48]	1	40	f	L3-L4	*C. lusitaniae*	Fluconazole	Debridement, discectomy without instrumentation	6	24
Chen et al., 2012 [49]	1	59	m	L3-L5	*C. parapsilosis*	Fluconazole	Debridement, discectomy with instrumentation	1.38	3
Grimes et al., 2012 [50]	1	63	f	L5-S1	*C. albicans*	Fluconazole, micafungin	Debridement	11	11
Jorge et al., 2012 [51]	1	73	m	L5-S1	*C. albicans*	Fluconazole, amphotericin B	Debridement, discectomy with instrumentation	6	NA
Joshi et al., 2012 [52]	1	63	m	T8-T9	*C. albicans*	Fluconazole, caspofungin	None	6.23	6
Theodoros et al., 2012 [53]	1	41	m	T11-T12	*C. albicans*	Caspofungin	None	1.38	10
Chen et al., 2013 [54]	1	41	m	L3-L4	*C. albicans*	Fluconazole	None	4.5	9
Ferrer Civeira et al., 2013 [55]	1	78	m	L4-L5	*C. tropicalis*	Fluconazole	None	NA	NA
Lebre et al., 2013 [56]	1	NA	NA	NA	NA	Amphotericin B	None	5	NA
Falakassa et al., 2014 [57]	1	58	f	T10-T12	*C. glabrata*	None	Debridement, discectomy with instrumentation	NA	NA
Iwata et al., 2014 [58]	3	56–72	3 m	1 L2-L3 2 L3-L4	3 *C. albicans*	Voriconazole, fluconazole	Debridement, discectomy without instrumentation	0.46–6.44	24–92
Oksi et al., 2014 [59]	1	37	m	L5-S1	*C. dubliniensis*	Fluconazole, amphotericin B	None	7.36	3
Savall et al., 2014 [60]	1	22	m	L1-L2	*C. albicans*	Fluconazole	None	2	NA
Oichi et al., 2015 [16]	1	79	m	L3-L4	*C. tropicalis*	Fluconazole, micafungin	None	NA	13
Salzer et al., 2015 [61]	1	47	m	L4-S1	*C. dubliniensis*	Fluconazole	None	3	NA
Zou et al., 2015 [62]	3	56–61	2 m 1 f	1 L3-L4 1 L3-L5 1 L3-S1	3 *C. albicans*	Amphotericin B	Debridement, discectomy with instrumentation	6–7	30–36
Mavrogenis et al., 2016 [63]	2	58–70	1 m 1 f	1 T3-T5 1 T10-T11	2 *C. albicans*	None	Debridement, discectomy with instrumentation	NA	0.69–24
Yu et al., 2016 [64]	1	70	m	L5-S1	*C. albicans*	Fluconazole	Debridement, discectomy with instrumentation	4	12
Lee et al., 2017 [65]	1	66	f	T11-T12	*C. albicans*	Fluconazole	Debridement, discectomy with instrumentation	6	NA
Stolberg-Stolberg et al., 2017 [10]	1	60	m	C4-C6	*C. albicans*	Fluconazole, caspofungin	Debridement, discectomy with instrumentation	7	24
Boyd et al., 2018 [66]	1	71	f	L5-S1	*C. albicans*	Fluconazole	Debridement	12.5	12
Che Saidi et al., 2018 [67]	1	67	f	L2-L3	*C. albicans*	None	Debridement, discectomy without instrumentation	NA	NA
Crane et al., 2018 [68]	1	27	m	T5-T7	*C. albicans*	Fluconazole, amphotericin B	Debridement, discectomy with instrumentation	5	48
El Khoury et al., 2018 [69]	1	53	f	C4-C5	*C. glabrata*	Voriconazole, micafungin	Debridement, discectomy with instrumentation	6	6
Gagliano et al., 2018 [70]	1	66	m	L3-L4	*C. glabrata*	Anidulafungin	Debridement, discectomy with instrumentation	NA	NA
Khoo et al., 2018 [8]	1	52	m	L4-L5	*C. parapsilosis*	Fluconazole, micafungin	Debridement, discectomy without instrumentation	6	3
Rambo et al., 2018 [71]	1	34	f	L5-S1	*C. albicans*	None	Debridement, discectomy with instrumentation	NA	15
Ruiz-Gaitán et al., 2018 [15]	2	42–66	2 m	NA	2 *C. auris*	Posaconazole, anidulafungin	None	NA	NA
Waldon et al., 2018 [72]	1	81	m	NA	*C. glabrata*	Anidulafungin	None	6	NA
Huang et al., 2019 [9]	1	32	m	C6-C7	*C. albicans*	Fluconazole, micafungin	Debridement, discectomy with instrumentation	12	3
Reaume et al., 2019 [73]	1	46	f	NA	*C. albicans*	Anidulafungin	None	NA	0
Timothy et al., 2019 [74]	1	54	NA	L3-L4	*C. glabrata*	None	Debridement, discectomy with instrumentation	NA	NA
Er et al., 2020 [75]	1	54	f	T12-L1	*C. parapsilosis*	Fluconazole	None	5	5
Overgaauw et al., 2020 [76]	1	78	m	L4-L5	*C. krusei*	Voriconazole, amphotericin B, anidulafungin	Debridement, discectomy with instrumentation	9	7
Relvas-Silva et al., 2020 [77]	1	66	f	T8-T9	*C. albicans*	Fluconazole, amphotericin B, anidulafungin	Debridement, discectomy with instrumentation	12	18
Supreeth et al., 2020 [78]	1	50	m	L4-L5	*C. auris*	Caspofungin	Debridement, discectomy with instrumentation	1.84	6
Upadhyay et al., 2020 [79]	1	61	m	L4-L5	*C. albicans*	Itraconazole, amphotericin B	Debridement, discectomy without instrumentation	3	NA
Lopes et al., 2021 [80]	1	72	m	T9-L4	*C. tropicalis*	Micafungin	Debridement, discectomy with instrumentation	2.76	NA
Moreno-Gomez et al., 2021 [81]	1	47	m	T8-T9	*C. albicans*	Fluconazole, amphotericin B	Debridement	11.5	8
von der Höh et al., 2021 [82]	1	73	m	L3-L4	*C. albicans*	Fluconazole	Debridement, discectomy with instrumentation	2.63	NA
Wajchenberg et al., 2021 [12]	1	69	m	L5-S1	*C. parapsilosis*	Fluconazole, anidulafungin	Debridement, discectomy with instrumentation	12	NA
Wang et al., 2021 [83]	5	55–72	4 m 1 f	2 L1-L2 1 L2-L3 1 L3-L4 1 L3-L5	5 *C. albicans*	Fluconazole, amphotericin B, caspofungin	Debridement, discectomy without instrumentation	NA	12–20
Yamada et al., 2021 [84]	1	74	m	L2-L3	*C. albicans*	Fluconazole	Debridement, discectomy with instrumentation	3.5	9
Duplan et al., 2022 [85]	1	50	m	L2-L3	*C. parapsilosis*	Fluconazole	Debridement	9	NA
Wang et al., 2022 [86]	1	62	f	L4-L5	*C. tropicalis*	Amphotericin B	Debridement, discectomy without instrumentation	12	30

**Table 2:** Baseline demographics and treatment outcome of 89 patients with *Candida* spondylodiscitis

	Available information	Data not available^†^
Age (years)*	61 (50, 68)	1 (1%)
Sex^†^		3 (3%)
Female	28 (33%)	
Male	58 (67%)	
Age-adjusted Charlson Comorbidity Index*	3 (1, 5)	5 (5%)
Immunosuppression^†^	18 (23%)	10 (11%)
Central venous line^†^	14 (18%)	13 (14%)
Previous spinal surgery^†^	14 (18%)	9 (10%)
Previous spinal surgery with instrumentation^†^	5 (6%)	9 (10%)
IV drug abuse^†^	12 (15%)	9 (10%)
Symptom duration until final diagnosis (weeks)*	9 (4, 17)	38 (42%)
Neck/back pain^†^	69 (99%)	19 (21%)
Fever/chills^†^	13 (19%)	21 (23%)
Weakness^†^	13 (19%)	21 (23%)
Paraplegia^†^	3 (4%)	19 (21%)
Leukocyte count (×10^9^/mL)*	8 (6, 10)	52 (58%)
C-reactive protein (mg/dL)*	3 (1, 6)	54 (60%)
Erythrocyte sedimentation rate (mm/h)*	65 (42, 96)	54 (60%)
CT-guided biopsy^†^	40 (53%)	14 (15%)
Spinal level affected^†^		6 (6%)
Cervical	6 (7%)	
Thoracic	19 (23%)	
Thoracolumbar	4 (5%)	
Lumbar	42 (51%)	
Lumbosacral	12 (14%)	
Candida strain^†^		1 (1%)
*C. albicans*	53 (60%)	
*C. auris*	3 (3%)	
*C. dubliniensis*	2 (2%)	
*C. glabrata*	6 (7%)	
*C. krusei*	3 (3%)	
*C. lusitaniae*	1 (1%)	
*C. parapsilosis*	6 (7%)	
*C. sake*	1 (1%)	
*C. tropicalis*	10 (11%)	
*Candida* spp.	3 (3%)	
Administration of empiric antibiotics^†^	29 (41%)	18 (20%)
Antifungal monotherapy^†^	46 (58%)	9 (10%)
Antifungals^†^		9 (10%)
Fluconazole	54 (68%)	
Voriconazole	6 (8%)	
Posaconazole	3 (4%)	
Itraconazole	2 (3%)	
Ketoconazole	1 (1%)	
Amphotericin B	30 (38%)	
Anidulafungin	7 (9%)	
Caspofungin	7 (9%)	
Micafungin	6 (8%)	
Flucytosine	3 (4%)	
Length of antifungal treatment (months)*	6 (3, 7)	25 (28%)
Surgical treatment regimen^†^		4 (4%)
No surgical intervention	27 (32%)	
Isolated debridement	11 (13%)	
Debridement and discectomy with instrumentation	29 (34%)	
Debridement and discectomy without instrumentation	18 (21%)	
Time to surgery since initial clinical presentation (weeks)*	5 (1, 13)	71 (79%)
Length of stay in hospital (weeks)*	9 (4, 13)	64 (71%)
Sepsis^†^	2 (3%)	10 (11%)
Revision surgery after initial treatment^†^	5 (6%)	10 (11%)
Final status^†^		14 (15%)
Full recovery	58 (74%)	
Partial recovery	11 (14%)	
Death	9 (12%)	
Follow-up (months)*	12 (6, 23)	31 (34%)

*Values are given as median and interquartile

^†^Values are given as absolute values and percentages

Among 85 patients with information on the *Candida* strain, 53 were for *Candida albicans* (62%) and 32 for *Candida non-albicans* (38%; Table 3). Both groups did not show statistically significant differences in baseline demographics, prior history, antifungal therapy, and surgical intervention. In contrast, patients with *Candida albicans* had a significantly higher leukocyte count (*p* = 0.039) and trended towards significantly increased ESR (*p* = 0.054) compared to those affected by *non-albicans Candida* spondylodiscitis. No statistically significant difference was noted in median follow-up, LOS, sepsis, and revision rate, as well as with respect to final recovery status and death. Likewise, the two year survivorship free of death was comparable between *albicans* (91%; 95% CI, 50 to 100%) *non-albicans Candida* (82%; 95% CI, 64 to 100%) spondylodiscitis (*p* = 0.68).

**Table 3:** Demographics and outcome stratified by *Candida albicans* versus *non-albicans Candida* strain

	*Candida albicans* (*n*=53)	*Non-albicans* (*n*=32)	*p*-value
Age (years)*	61 (46, 67)	60.5 (50, 69)	0.40
Sex^†^			0.81
Male	35 (67%)	22 (71%)	
Female	17 (33%)	9 (29%)	
Age-adjusted Charlson Comorbidity Index*	3 (1, 5)	3 (1, 6)	0.66
Previous spinal surgery^†^	10 of 48 (21%)	4 of 31 (13%)	0.55
Previous spinal surgery with instrumentation^†^	2 of 48 (4%)	3 of 31 (10%)	0.38
Leukocyte count (×10^9^/mL)*	9 (6.95, 11.65)	6.5 (3.96, 8)	**0.039**
C-reactive protein (mg/dL)*	3.4 (2.18, 5.685)	1.5 (0.7, 11.8)	0.28
Erythrocyte sedimentation rate (mm/h)*	87 (62, 109)	51 (41, 66)	**0.054**
Spinal level affected^†^			0.98
Cervical	4 (8%)	2 (7%)	
Thoracic	12 (23%)	6 (21%)	
Thoracolumbar	2 (4%)	2 (7%)	
Lumbar	26 (50%)	14 (50%)	
Lumbosacral	8 (15%)	4 (14%)	
Candida strain^†^			
*C. auris*		3 (9%)	
*C. dubliniensis*		2 (6%)	
*C. glabrata*		6 (19%)	
*C. krusei*		3 (9%)	
*C. lusitaniae*		1 (3%)	
*C. parapsilosis*		6 (19%)	
*C. sake*		1 (3%)	
*C. tropicalis*		10 (31%)	
Antifungal monotherapy^†^	28 of 48 (58%)	17 of 28 (61%)	0.99
Antifungal class if monotherapy^†^			0.36
Azole	21 (75%)	10 (59%)	
Echinocandin	2 (7%)	4 (24%)	
Amphotericin B	5 (18%)	3 (18%)	
Length of antifungal treatment (months)*	6 (3.22, 8.28)	5.5 (2.76, 7.36)	0.51
Surgical intervention^†^	38 of 53 (72%)	18 of 29 (62%)	0.46
Follow-up (months)*	12 (8, 24)	7.5 (5, 19)	0.13
Length of stay in hospital (weeks)*	9 (3, 13)	7.5 (4, 12)	0.86
Sepsis^†^	1 of 50 (2)	1 of 28 (4%)	0.67
Revision surgery after initial treatment^†^	3 of 50 (6)	1 of 28 (4%)	0.64
Final status^†^			0.36
Full recovery	37 (77%)	20 (69%)	
Partial recovery	5 (10%)	6 (21%)	
Death	6 (13%)	3 (10%)	

Numbers in bold indicate reaching significance (*p*-value < 0.05)

*Values are given as median and interquartile

^†^Values are given as absolute values and percentages

In total, 76 patients had a known survival status at the last follow-up, with 67 surviving and 9 dying by disease (Table 4). Younger age (*p* = 0.042) and longer length of antifungal therapy (*p* = 0.061) were predictive of survival, whereas outcome did not differ based on *Candida* strain (*p* = 0.74) and affected spinal level (*p* = 0.44).

**Table 4:** Patient demographics stratified by survival following *Candida* spondylodiscitis

	Survival (*n*=67)	Death (*n*=9)	*p*-value
Age (years)*	59 (47, 67)	67 (62, 72)	**0.042**
Sex^†^			0.99
Male	43 (65%)	6 (67%)	
Female	23 (35%)	3 (33%)	
Age-adjusted Charlson Comorbidity Index*	3 (1, 4)	4 (3, 5.5)	0.14
Previous spinal surgery^†^	51 of 64 (80%)	9 of 9 (100%)	0.35
Previous spinal surgery with instrumentation^†^	59 of 64 (92%)	9 of 9 (100%)	0.99
Leukocyte count (×10^9^/mL)*	8.2 (6.6, 10.8)	6.8 (5.6, 8)	0.37
C-reactive protein (mg/dL)*	3.145 (1.5, 6.07)	1.1 (1.1, 1.1)	0.28
Erythrocyte sedimentation rate (mm/h)*	68 (41, 98)	51 (51, 51)	0.61
Spinal level affected^†^			0.44
Cervical	6 (9%)	0 (0%)	
Thoracic	15 (23%)	3 (38%)	
Thoracolumbar	3 (5%)	1 (13%)	
Lumbar	30 (45%)	4 (50%)	
Lumbosacral	12 (18%)	0 (0%)	
Candida strain^†^			0.74
*C. auris*	1 (4%)	0 (0%)	
*C. dubliniensis*	2 (9%)	0 (0%)	
*C. glabrata*	3 (13%)	0 (0%)	
*C. krusei*	2 (9%)	1 (33%)	
*C. lusitaniae*	1 (4%)	0 (0%)	
*C. parapsilosis*	6 (26%)	0 (0%)	
*C. sake*	1 (4%)	0 (0%)	
*C. tropicalis*	7 (30%)	2 (67%)	
Antifungal monotherapy^†^	28 of 63 (44%)	2 of 7 (29%)	0.69
Antifungal class if monotherapy^†^			0.11
Azole	25 (71%)	2 (40%)	
Echinocandin	3 (9%)	2 (40%)	
Amphotericin B	7 (20%)	1 (20%)	
Length of antifungal treatment (months)*	6 (3.22, 8.5)	2.695 (2.005, 4.38)	**0.061**
Surgical intervention^†^	22 of 67 (33%)	4 of 9 (44%)	0.48
Follow-up (months)*	12 (6, 24)	8 (.69, 13)	0.15

Numbers in bold indicate reaching significance (*p*-value < 0.05)

*Values are given as median and interquartile

^†^Values are given as absolute values and percentages

## Discussion

Limited remains known on *Candida* spondylodiscitis outside of case reports and smaller case series. As such, this systematic review analyzed 89 patients treated for spondylodiscitis at a median follow-up of 12 months. Our results demonstrated one in five patients affected by *Candida* spondylodiscitis to die within two years. Importantly, *Candida albicans* and *non-albicans* were similar in their prognosis, whereas younger age and prolonged antifungal treatment were associated with increased survival.

### Baseline clinical and demographic factors

Knowledge of baseline demographics among patients affected by *Candida* spondylodiscitis is important, as it may allow to identify potential patients at risk. First reports date back to the 1970s [87], and *Candida* spondylodiscitis has since then been associated with immunocompromised patients, and those with other significant comorbidities including diabetes, obesity, or IV drug abuse [88–90]. In this systematic review, we identified most affected patients to be males in their sixties, with one in four demonstrating signs of immunocompromise, and another 15% reporting previous IV drug use. While these findings partially reflect the aforementioned historical literature, one must acknowledge that the majority of patients had no clearly attributable risk factors. Prospective multicentre studies will be necessary to draw final conclusions, based on comparison with spondylodiscitis caused by other pathogens.

### Diagnostic challenges of *Candida* spondylodiscitis

The diagnosis of *Candida* spondylodiscitis remains challenging, especially as symptom onset is subacute, and oftentimes gradually progressive over weeks to months [91–93]. In fact, 99% of patients had back or neck pain during initial clinical assessment, while less than 20% had systematic signs of infection (fever). Correspondingly, and similar to previous findings, CRP was only mildly elevated [94–96]. Although initial identification of *Candida* spondylodiscitis may therefore remain difficult, a number of factors distinguish it from a bacterial entity, once the diagnosis of spondylodiscitis is suspected. Foremost, the lumbar spine is more commonly affected (50%) [97], symptom onset is less acute, and *Candida* spondylodiscitis rarely causes chills [98, 99]. This differentiation is important, as it may allow for an earlier antifungal treatment initiation.

### Antifungal treatment and surgical considerations as the mainstay of therapy

Pathogen eradication is the primary goal in treatment of *Candida* spondylodiscitis, with its success depending on rates of antifungal disc penetration and antimicrobial resistances [76]. The Infectious Diseases Society of America (IDSA) recommends fluconazole (400 mg; 6 mg/kg daily) for six to 12 months in case of *Candida osteomyelitis* but does not specify for spondylodiscitis [100]. Although our findings partially reflect current IDSA recommendations on *Candida osteomyelitis*, treatment still varied significantly among the 89 patients. We believe this heterogeneity to be attributable to possible treatment resistances requiring changes in antifungal medications [101], as well as contradictory recommendations on antifungal length, ranging from a few weeks to over a year [12, 24, 53]. Finally, different approaches on empiric treatment may have been a contributing factor [29, 54, 61]. Despite these discrepancies, antimicrobial therapy remains the most important part of the treatment of spondylodiscitis [5] with surgery only being recommended in cases of spinal instability, a mass effect due to abscess, or neurologic deterioration [10, 102, 103]. We believe the prolongated symptom onset prior to definite antifungal treatment, combined with possible extensive local disease progression, and possibly unclear preoperative diagnosis to explain the high rates of surgery among our patients (68%). Despite an increase in antifungal resistance, large-scale surveys of pathogenic yeasts isolated from blood cultures suggest that clinical consequences may not be expected from this trend [104]. Further, Candida spp. may be subject to a confirmation bias owing to advances in testing susceptibility [105]. Surgical intervention was most commonly pursued if spinal instability was suspected or management solely based on antifungal agents deemed insufficient.

### Prognostic factors in Candida spondylodiscitis

The prognosis in *Candida* spondylodiscitis remained poor, with only 77% achieving full recovery, and 12% dying at a median follow-up of one year. The two year calculated survivorship free of death was even as low as 80%, predicting one in five patients to die within two years of diagnosis. Importantly, *Candida* strain was not predictive of death, as previously described in periprosthetic joint infections [16, 40]. In contrast, younger age at diagnosis and longer antifungal treatment were predictive of survival, with the latter being a promising alternative for future research. Of note, only 6% of patients were in need of revision surgery. This is important, as spinal revision surgery is known to significantly compromise functional recovery [77, 86, 106].

### Limitations

This systematic review had limitations that in return were attributable to the weaknesses of its included studies. Foremost, most articles included five cases or less, limiting the overall number and generalizability. In addition, not all information could be included for every patient. In specific, details on secondary diseases were not provided in the majority of articles, and follow-up defined inconsistently. Additionally, some data such as clinical examination results and laboratory results were inconsistently reported among cases. This may have compromised the generalizability of our results. We have nevertheless included these data points to enhance to informative value of our tables, even when data could not be included in our statistical analysis. Finally, we were not able to provide regression models to precise potential risk and outcome factors, as the high proportion of missing information would have corrupted the analysis.

In conclusion, patients affected by *Candida* spondylodiscitis tend to be males in their sixties, present with local rather than systematic symptoms, and have a poor short-term prognosis with one in five dying within two years of diagnosis. This first systematic review on *Candida* spondylodiscitis might help physicians in identifying patients at risk and when providing a prognosis. Consensus meetings will be necessary to determine an optimal future treatment approach.

### Supplementary information

ESM 1 (DOCX 29 kb)

## Data Availability

The authors declare that data will be made available upon reasonable request.

## Code availability (software application or custom code)

The authors declare that code for data analysis will be made available upon reasonable request.
