# Supplementary material for: Candida spondylodiscitis: a systematic review and meta-analysis of seventy two studies
Source: Int Orthop. 2023 Oct 4;48(1):5–20. doi: 10.1007/s00264-023-05989-2 (PMC10766661; doi:10.1007/s00264-023-05989-2)
Supplement: Supplementary file 1 — (DOCX 29 kb) [file 264_2023_5989_MOESM1_ESM.docx]

Candida Spondylodiscitis: A Systematic Review and Metanalysis of 89 Cases

# APPENDIX

## Supplementary Table 1. Databases queried and search terms used

| Database | Search query | Results retrieved |
| --- | --- | --- |
| PubMed | (candida) AND (spondylodiscitis) | 99 |
| Web of Science | candida (All Fields) AND spondylodiscitis (All Fields) | 84 |
| Embase | ('candida'/exp OR candida) AND ('spondylodiscitis'/exp OR spondylodiscitis) | 192 |
| Scopus | ( ALL ( candida ) AND ALL ( spondylodiscitis ) ) | 584 |
| OVID Medline | (candida and spondylodiscitis) | 79 |
|  |  | Total: 1038 |

## Supplementary Table 2. PICO criteria

| Item | Description |
| --- | --- |
| Population | Patients with laboratory-confirmed spondylodiscitis caused by *Candida spp.* |
| Intervention | Treatment of Candida spondylosdiscitis involves antifungal agents. Neurologic deterioration, spinal instability, or compression of the spinal cord or nerve roots may necessitate surgical intervention. |
| Comparator | We compare treatment strategies including type of antifungals used and if surgical debridement. Since a confirmation of Candida spondylodiscitis warrants treatment, no control group was available to compare treatment versus no-treatment. |
| Outcome(s) | Primary outcome of interest of this study was full recovery following treatment of Candida spondylodiscitis.  Secondary outcomes include diagnostic workup by CT-guided biopsy, laboratory variables including leukocyte count, CRP, and ESR; treatment regiment decisions including choice of antifungal agents and surgical intervention. We also collected data on revision surgery, re-infection, and complications. |
